# GINS2 promotes oral squamous cell carcinoma progression and immune evasion by recruiting PD-L1^+^ neutrophils and modulating the PTP4A1/PKM2 axis

**DOI:** 10.3389/fimmu.2025.1637296

**Published:** 2025-11-05

**Authors:** Bo Wei, Jiajia Yin, Chuyan Shi, Hui Sun, Bing Liu, Peng Chen

**Affiliations:** 1 Department of Stomatology, The First Medical Center of Chinese PLA General Hospital, Beijing, China; 2 Department of Anesthesiology, The First Medical Center of Chinese PLA General Hospital, Beijing, China; 3 Medical School of Chinese PLA, Beijing, China; 4 Department of Radiology, The First Medical Center of Chinese PLA General Hospital, Beijing, China; 5 Department of Stomatology, Air Force Medical Center of Chinese PLA Air Force Medical University, Beijing, China

**Keywords:** GINS2, oral squamous cell carcinoma (OSCC), tumor microenvironment, neutrophils, immune evasion, PTP4A1, PD-L1, tumor-associated neutrophils (TANs)

## Abstract

**Introduction:**

The GINS complex subunit 2 (GINS2) is crucial for DNA replication, but its specific roles in oral squamous cell carcinoma (OSCC) pathogenesis and tumor microenvironment (TME) modulation are poorly defined.

**Methods:**

GINS2 expression was analyzed using TCGA data and validated in OSCC patient tissues and cell lines via qPCR, Western blot (WB), and immunohistochemistry (IHC). Functional assays (CCK-8, colony formation, wound healing, Transwell invasion) and *in vivo* xenograft models assessed the impact of GINS2 knockdown (sh-GINS2) or overexpression (OE-GINS2) on OSCC cell behavior and tumorigenesis. Mechanistic links involving PTP4A1 and PKM2 were explored using Co-immunoprecipitation (Co-IP) and immunofluorescence (IF). Immune correlations were assessed in TCGA/TIMER2.0 (PDCD1, LAG3, CTLA4, HAVCR2), and PD-1/TIM-3 on CD8^+^ T cells were quantified by flow cytometry in co-culture. Neutrophil features (PD-L1 expression) and interventions (neutrophil depletion, anti-PD-L1) were evaluated *in vitro* and immune-reconstituted *in vivo* settings.

**Results:**

GINS2 was significantly upregulated in OSCC tissues and cell lines, correlating with advanced clinical stage and higher pathological grade. GINS2 knockdown suppressed proliferation, colony formation, migration, and invasion *in vitro*, and inhibited tumor growth *in vivo*. At the protein level, GINS2 physically associated with PTP4A1 and monotonically modulated its steady-state abundance; PTP4A1 interacted and co-localized with PKM2. In TCGA, GINS2 expression positively correlated with T-cell exhaustion markers, and altering GINS2 in OSCC cells changed PD-1 and TIM-3 on co-cultured CD8^+^ T cells. GINS2 expression also correlated with neutrophil infiltration; GINS2 overexpression increased tumor-associated neutrophils (TANs) *in vivo*, and Ly6G neutrophil depletion attenuated GINS2-driven tumor enhancement. OSCC-associated neutrophils exhibited elevated PD-L1 expression, correlating positively with GINS2 levels. GINS2 knockdown sensitized OSCC models to anti-PD-L1 therapy, reducing tumor growth and Ki67 expression, particularly when combined with T cells and neutrophils.

**Discussion:**

GINS2 acts as a key oncogenic driver in OSCC, promoting tumor progression and facilitating immune evasion. Its effects appear to involve a proximal GINS2–PTP4A1–PKM2 module and the recruitment/polarization of PD-L1^+^ neutrophils linked to T-cell dysfunction. Targeting the GINS2 axis—potentially in combination with PD-L1 blockade—warrants further investigation in OSCC, with downstream signaling mechanisms to be clarified in future work.

## 1 Introduction

Oral squamous cell carcinoma (OSCC) represents a predominant subtype of head and neck cancer, posing a substantial global health challenge due to its aggressive biological behavior and often unfavorable clinical outcomes (1). Despite advances in multimodality treatment approaches combining surgery, radiotherapy, and chemotherapy, the 5-year overall survival rates for patients diagnosed with advanced-stage OSCC have shown only modest improvement over recent decades, frequently lingering below 50-60% (2, 3). This sobering statistic is largely attributable to the intrinsic propensity of OSCC for rapid local invasion, early dissemination to regional lymph nodes, and the eventual development of distant metastases (4, 5). Furthermore, the efficacy of systemic therapies is often hampered by the emergence of intrinsic or acquired resistance mechanisms, underscoring the urgent need for a more profound understanding of the molecular underpinnings driving OSCC progression and therapeutic failure (6). Identifying novel molecular drivers and therapeutic vulnerabilities is therefore paramount for developing more effective treatment strategies.

The GINS complex, comprising four subunits (Sld5, Psf1, Psf2, Psf3 – encoded by *GINS1–4* respectively), is an essential component of the eukaryotic replisome, playing a critical role in the initiation and elongation phases of DNA replication (7). GINS forms the core of the CMG (Cdc45-MCM-GINS) helicase, which unwinds DNA at the replication fork. Given its fundamental role in DNA synthesis (8), dysregulation of GINS complex components has been increasingly implicated in tumorigenesis, likely by facilitating uncontrolled cell proliferation (9, 10). Specifically, GINS2 (Psf2) overexpression has been reported in various human malignancies, including head and neck squamous cell carcinomas (HNSCC) (11), epithelial ovarian cancer (12), Osteosarcoma (13), cervical cancer (14), often correlating with aggressive tumor characteristics and poor patient prognosis. While these studies highlight GINS2 as a potential oncogene, its specific functional contributions and downstream signaling pathways within the complex molecular landscape of OSCC remain largely unexplored.

The tumor microenvironment (TME) plays a crucial role in OSCC development, progression, and response to therapy (15). A key feature of the OSCC TME is profound immune dysregulation, enabling tumor cells to evade host anti-tumor immunity. This involves multiple mechanisms, including the recruitment of immunosuppressive cell populations and the upregulation of immune checkpoint molecules, such as Programmed Death-Ligand 1 (PD-L1), which induces T cell exhaustion and anergy (16). While cytotoxic CD8+ T cells are critical for controlling tumor growth, their function is often impaired in OSCC due to chronic antigen exposure and the suppressive TME, leading to an exhausted phenotype characterized by reduced effector function and sustained expression of inhibitory receptors like PD-1, LAG3, and TIM3 (17).

Neutrophils, traditionally viewed as first responders in acute inflammation, are increasingly recognized as versatile players within cancer, adopting context-dependent anti-tumor (N1) or pro-tumor (N2) programs shaped by cytokines, hypoxia, and tumor-derived cues (18, 19). In many established cancers, including OSCC, tumor-associated neutrophils (TANs) frequently assume pro-tumor functions that support angiogenesis, invasion, metastasis, and immune suppression—via arginase-1, reactive oxygen species, neutrophil extracellular traps (NETs), and checkpoint-like ligands (19, 20). Recent single-cell work further shows deterministic reprogramming of neutrophils within tumors, indicating malignant signals can imprint neutrophil fate and function *in situ* (21). In head-and-neck/OSCC settings specifically, PD-L1^+^ TANs restrain T-cell activity through the PD-1 pathway and can be induced by factors such as GM-CSF (22, 23). This duality provides the conceptual basis for our hypothesis that tumor-intrinsic GINS2 not only drives proliferation but also recruits and polarizes TANs toward immunosuppressive states that blunt CD8^+^ T-cell function in OSCC.

GINS2 is overexpressed in head-and-neck squamous cell carcinoma and promotes tumor progression by altering RRM2 expression (11), and systems analyses nominate GINS2 as an upstream modulator of metastatic programs in HNSCC (24). Mechanistically, GINS2 regulates cancer-cell proliferation via the phosphatase PTP4A1 (PRL-1), establishing a direct GINS2→PTP4A1 link (25). PTP4A1 drives oncogenic signaling through PI3K/AKT and EMT (26) and, in OSCC specifically, promotes mitochondrial metabolic reprogramming with concordant increases in PKM2 transcription (27). Consistently, PKM2 is elevated in OSCC and associates with aggressive clinicopathologic features and poor prognosis (28), and PKM2 also exerts non-glycolytic signaling functions that influence cell fate decisions (29). On the immune axis, tumor-infiltrating PD-L1^+^ neutrophils suppress T-cell function in head and neck cancer via GM-CSF–driven programs (23), and the PD-L1/PD-1 checkpoint directly restrains neutrophil cytotoxicity in cancer (22); in OSCC, neutrophils further promote tumor progression through Chemerin–JAK2/STAT3 signaling (30). In parallel, primary tissue and single-cell studies document CD8^+^ T-cell exhaustion programs in OSCC with sustained inhibitory-receptor expression (16). Collectively, these primary data motivate testing a GINS2→PTP4A1/PKM2 pathway that couples tumor-intrinsic proliferation/metabolism to PD-L1^+^ neutrophil–mediated T-cell dysfunction in the OSCC microenvironment.

Given the established role of GINS2 in proliferation and its overexpression in multiple cancers, coupled with the critical influence of the immune microenvironment in OSCC, we hypothesized that GINS2 functions as a key driver of OSCC progression by not only promoting tumor cell growth and invasion but also by actively shaping an immunosuppressive TME. We further postulated that GINS2 might interact with signaling molecules like PTP4A1 and PKM2 and influence the recruitment and function of immune cells, particularly neutrophils expressing PD-L1. Therefore, this study aimed to: (1) comprehensively evaluate GINS2 expression in OSCC and its correlation with clinicopathological features and prognosis; (2) determine the functional impact of GINS2 on OSCC cell proliferation, migration, invasion, and *in vivo* tumorigenicity; (3) investigate potential interactions between GINS2, PTP4A1, and PKM2; (4) analyze the association of GINS2 with T cell exhaustion markers and neutrophil infiltration/function (PD-L1 expression) in the OSCC TME; and (5) assess whether targeting the GINS2 pathway, alone or in combination with immune checkpoint blockade, holds therapeutic potential for OSCC.

## 2 Materials and methods

### 2.1 Bioinformatics analysis

Clinical transcriptomic data and associated clinical information for OSCC were acquired from The Cancer Genome Atlas (TCGA) database portal (https://portal.gdc.cancer.gov/), specifically focusing on the Head and Neck Squamous Cell Carcinoma (HNSC) cohort with appropriate filtering for oral cavity sites. Raw RNA sequencing reads (FASTQ files) were aligned to the human reference genome (GRCh38), and gene expression levels were quantified as Transcripts Per Million (TPM) or using featureCounts (v2.0; Subread package; http://subread.sourceforge.net/) to obtain raw counts. Differential expression gene (DEG) analysis comparing tumor versus adjacent normal tissues was conducted using the R package DESeq2 (v3.1.0; https://bioconductor.org/packages/release/bioc/html/DESeq2.html) on the raw counts. DEGs were identified based on criteria of |log2 Fold Change| > 2 and False Discovery Rate (FDR) < 0.05. Volcano plots were generated using the ggplot2 R package to visualize DEGs. Pan-cancer expression analysis of GINS2 across various TCGA tumor types and corresponding normal tissues was performed using integrated online tools (e.g., GEPIA2, UALCAN) or custom scripts plotting TCGA TPM data. The complete tumor–normal DEG results (log_2_FC and FDR for all genes) are provided in **Supplementary Table 1**.

### 2.2 Protein-protein interaction network construction

For the PPI analysis, we used as seeds the intersection of differentially expressed genes (tumor vs. adjacent normal; |log_2_FC| > 2; FDR < 0.05 by DESeq2) and overall-survival–associated genes (log-rank P < 0.05) from the TCGA oral-cavity subset, queried in STRING v12.0 (Homo sapiens; minimum required interaction score > 0.4; other settings default). The resulting network was exported and visualized in Cytoscape v3.9.1; node size/color encode connectivity (degree), edge thickness reflects the STRING combined score, and hub metrics were computed with Cytoscape’s network analysis. Note: PTP4A1 did not meet the DEG cutoff, and PKM is quantified at the gene level in TCGA (collapsing PKM1/PKM2), which can under-represent isoform-specific PKM2 changes.

### 2.3 Patients

A total of 80 patients presenting with primary OSCC who underwent surgical treatment between January 2020 and December 2024 were retrospectively included in this study. Paired adjacent healthy oral mucosal tissues were also collected from the same patients where feasible. Inclusion criteria were: histologically confirmed primary OSCC both genders, aged 18–65, no prior anti-cancer therapy (chemotherapy, radiotherapy, or immunotherapy), follow-up of more than 12 months, availability of tissue samples and complete clinical records. Exclusion criteria included: recurrent OSCC, history of other malignancies, radiation in the head and neck area or a history of chemotherapy or antibody therapy. This investigation was approved by the internal ethics committee of Chinese PLA General Hospital (Approval No. S2025-018-01) and was conducted according to the principles outlined in the Declaration of Helsinki. All subjects provided written informed consent prior to participation. Tumor pathological grade was classified based on the World Health Organization (WHO) classification criteria by experienced pathologists. Clinical stage information was retrieved from patient records.

Patients were enrolled using a consecutive sampling approach from oral-cavity subsites (tongue, floor of mouth, gingiva, buccal mucosa, hard palate; lip and oropharynx excluded). Cases with incomplete key variables (age, sex, site, stage, treatment history) were excluded *a priori*; no data imputation was performed. Pathology review and IHC scoring were performed blinded to clinical outcomes. For correlative analyses (e.g., GINS2 vs PD-L1 MFI on neutrophils), we prespecified inclusion of only matched tumor–blood pairs collected within 24 h of surgery and processed with identical workflows. Group stratification into High- vs Low-GINS2 used the median H-score within the study cohort and was applied only to patient tissue analyses (not animal studies).

### 2.4 Cell lines

Human OSCC cell lines (Cal27, HN6, SCC4, SCC25) and normal human oral keratinocytes (HOK) were purchased from the American Type Culture Collection (ATCC, Manassas, VA, USA). Cell lines were authenticated using short tandem repeat (STR) profiling upon receipt and periodically thereafter. The cell lines were cultured and maintained in Dulbecco’s Modified Eagle Medium (DMEM; Gibco, USA) supplemented with 10% fetal bovine serum (FBS; Gibco Laboratories, USA) and 1% penicillin/streptomycin solution (Gibco) at 37°C in a humidified incubator containing 5% CO2.

### 2.5 Cell culture and transfection

For gene knockdown experiments, short hairpin RNA (shRNA) constructs targeting human GINS2 (sh-GINS2: 5’-GATTAACCTGAAACAAAGA-3’) and a non-targeting negative control shRNA (sh-NC: 5’-TTTCTCCGAACGTGTCACGT-3’) cloned into appropriate vectors (pLKO.1) were obtained from commercial sources (Sigma-Aldrich or synthesized by GenePharma). For overexpression experiments, a GINS2 expression plasmid (OE-GINS2) containing the full-length human GINS2 cDNA under a constitutive promoter (CMV in pcDNA3.1) and a corresponding empty vector control (OE-NC) were used. Transient transfections into OSCC cell lines (HN6, SCC25) were performed using Lipofectamine 3000 reagent (Invitrogen, Carlsbad, CA, USA) according to the manufacturer’s instructions. Cells were typically harvested 48–72 hours post-transfection for subsequent experiments. For stable cell line generation, lentiviral transduction followed by selection (puromycin) was employed where necessary (for *in vivo* studies). Knockdown or overexpression efficiency was confirmed by qPCR and Western blot.

### 2.6 Real-time quantitative PCR analysis

Total cellular RNA was isolated from cultured cells using TRIzol reagent (Invitrogen) or a comparable RNA isolation kit according to the manufacturer’s protocol. First-strand complementary DNA (cDNA) synthesis was performed using 1-2 μg of total RNA with the PrimeScript RT Reagent Kit (Takara Bio, Shiga, Japan). Real-time quantitative PCR amplification was carried out using SYBR Green PCR Master Mix (Takara Bio) on a suitable qPCR system (Applied Biosystems 7500). The specific primer sequences used were: GINS2 Forward: 5’-AGCCAAACTCCGAGTGTCTGCT-3’; GINS2 Reverse: 5’-CTTGTGTGAGGAAAGTCCCGCT-3’; β-Actin Forward: 5’-CACCATTGGCAATGAGCGGTTC-3’; β-Actin Reverse: 5’-AGGTCTTTGCGGATGTCCACGT-3’. Thermal cycling conditions typically involved an initial denaturation step, followed by 40 cycles of denaturation, annealing, and extension. Relative quantification of gene expression was calculated using the 2-ΔΔCt method, with β-actin serving as the endogenous control for normalization.

### 2.7 Western blot analysis

Cells were lysed in RIPA buffer (radioimmunoprecipitation assay buffer) supplemented with protease and phosphatase inhibitor cocktails (Sigma-Aldrich or Roche). Protein concentrations were determined using the BCA Protein Assay Kit (Beyotime, Shanghai, China). Equal amounts of protein (typically 20-40 μg per lane) were separated by sodium dodecyl sulfate-polyacrylamide gel electrophoresis (SDS-PAGE) on 10-12% gels and subsequently transferred onto polyvinylidene difluoride (PVDF) or nitrocellulose membranes (Bio-Rad, Hercules, CA, USA). Following transfer, membranes were blocked for 1 hour at room temperature with 5% nonfat dry milk or bovine serum albumin (BSA) dissolved in Tris-buffered saline containing 0.1% Tween-20 (TBST). Membranes were then incubated overnight at 4°C with primary antibodies diluted in blocking buffer. The primary antibodies used were: anti-GINS2 (Abcam, ab197123; dilution 1:1000), anti-PTP4A1 (also known as PRL-1; Abcam, ab168643; 1 µg/ml), anti-PKM2 (Abcam, ab120577; dilution 1:1000 or Cell Signaling Technology #4053, 1:1000), and anti-β-actin (Abcam, ab8227; dilution 1:3000) as a loading control. After washing thoroughly with TBST, membranes were incubated with appropriate horseradish peroxidase (HRP)-conjugated secondary antibodies (anti-rabbit IgG HRP-linked antibody, Abcam ab6721; dilution 1:2500) for 1–2 hours at room temperature. Protein bands were visualized using an enhanced chemiluminescence (ECL) detection kit (Thermo Fisher Scientific, Inc., Waltham, MA, USA) and imaged using a suitable chemiluminescence detection system (Bio-Rad ChemiDoc). Band intensities were quantified using ImageJ software (NIH, Bethesda, MD, USA) or similar software, normalizing target protein levels to the corresponding β-actin loading control.

### 2.8 Immunohistochemistry staining

Formalin-fixed, paraffin-embedded (FFPE) OSCC tumor tissue sections (4 μm thickness) were deparaffinized in xylene and rehydrated through a graded series of ethanol solutions. Antigen retrieval was performed by heating slides in an appropriate buffer (citrate buffer pH 6.0 or Tris-EDTA buffer pH 9.0) using a microwave or pressure cooker. Endogenous peroxidase activity was quenched by treating sections with 3% hydrogen peroxide in methanol for 20 minutes. After blocking non-specific binding sites with normal goat serum for 30–60 minutes at room temperature, sections were incubated overnight at 4°C with primary antibodies: anti-GINS2 (Abcam, ab197123; dilution 1:500), anti-MPO (Myeloperoxidase; Abcam, ab208670; dilution 1:1000), or anti-Ki67 (Abcam, ab279653; dilution 1:1000). Following washes, sections were incubated with an HRP-polymer anti-rabbit/mouse secondary antibody kit (Fuzhou Maixin Biotech, Fuzhou, China) according to the manufacturer’s instructions. Visualization was achieved using a 3,3’-Diaminobenzidine (DAB) substrate kit (Fuzhou Maixin Biotech), resulting in a brown precipitate at the antigen site. Sections were counterstained with hematoxylin to visualize nuclei. Finally, samples were dehydrated through an ethanol gradient, cleared with xylene, and mounted using neutral gum. Images were captured using a light microscope (Olympus or Nikon) equipped with a digital camera. Quantification of staining intensity or percentage of positive cells was performed using ImageJ. GINS2 staining was quantified as an H-score (intensity 0–3 × % positive, range 0–300) by two pathologists blinded to clinical data. Tumors were classified as High-GINS2 (H-score ≥ median) or Low-GINS2 (H-score < median) for groupwise comparisons. These groups are used only for patient tissue analyses.

### 2.9 Immunofluorescence staining

For immunofluorescence analysis of tissue sections or cultured cells grown on coverslips, samples were fixed with 4% paraformaldehyde (PFA) for 15–20 minutes, followed by permeabilization with 0.2% Triton X-100 in PBS for 10 minutes. Non-specific binding was blocked with 2-5% BSA or normal serum in PBS for 30–60 minutes at room temperature. Samples were then incubated with primary antibodies overnight at 4°C. The primary antibodies used included: anti-GINS2 (Proteintech, #16247-1-AP, 1:1000), anti-CD8 (Servicebio, #GB13068, 1:500), anti-Myeloperoxidase (MPO; R&D Systems, #AF3667, concentration specified by manufacturer or 1:200), anti-citrullinated Histone H3 (CitH3; Abcam, #ab5103, 1:500), anti-PTP4A1 (Proteintech, #67584-1-Ig, 1:200), anti-PKM2 (Cell Signaling Technology, #4053T, 1:200), anti-PD-1 (Cell Signaling Technology, #86163, 1:200), and anti-TIM-3 (Cell Signaling Technology, #75743, 1:200). After washing, sections were incubated with species-specific secondary antibodies conjugated to Alexa Fluor dyes (Alexa Fluor 488 goat anti-rabbit IgG, Abcam ab150077; Alexa Fluor 594 goat anti-mouse IgG) for 1–2 hours at room temperature, protected from light. Nuclei were counterstained with DAPI (4’,6-diamidino-2-phenylindole). Coverslips were mounted onto glass slides using an anti-fade mounting medium (ProLong Gold, Invitrogen). Images were acquired using a fluorescence microscope or confocal laser scanning microscope (Leica or Zeiss). Quantification of fluorescence intensity or co-localization was performed using appropriate software (ImageJ with Coloc 2 plugin). IF signal for GINS2 and CD8 was quantified in ImageJ (mean integrated density normalized to area and DAPI). Samples were grouped as High-GINS2 or Low-GINS2 using the median H-score from matched sections.

### 2.10 Cell viability assay

Cell viability was assessed using the Cell Counting Kit-8 (CCK-8; Dojindo Laboratories, Kumamoto, Japan). Transfected OSCC cells (HN6, SCC25) were seeded into 96-well plates at a density of approximately 3,000-5,000 cells per well in 100 μL of complete medium. At specified time points (e.g., 0, 24, 48, 72, 96 hours), 10 μL of CCK-8 solution was added to each well, followed by incubation for 1–4 hours at 37°C according to the manufacturer’s protocol. The absorbance at 450 nm was measured using a microplate spectrophotometer (Thermo Fisher Scientific Varioskan). Viability was typically expressed relative to the initial time point or control group.

### 2.11 Colony formation assays

For colony formation assays, transfected OSCC cells (HN6, SCC25) were seeded at a low density (e.g., 500–1000 cells per well) into 6-well plates and cultured in complete medium for approximately 10–14 days, with medium changes every 3–4 days, until visible colonies formed. At the end of the incubation period, colonies were washed with PBS, fixed with 4% paraformaldehyde for 15–30 minutes, and stained with 0.1% crystal violet solution for 20–30 minutes. After washing excess stain with water and air-drying, the plates were photographed. The number of colonies (typically defined as >50 cells) in each well was counted manually or using imaging software (ImageJ).

### 2.12 EdU assay

CD8+ T cell proliferation was assessed using the Cell-Light EdU Apollo567 *In Vitro* Kit (RiboBio, Guangzhou, China) or similar assay. Purified CD8+ T cells, co-cultured under different conditions (with neutrophils, +/- anti-PD-L1), were incubated with 50 μM EdU for 2–4 hours. Cells were then harvested, fixed with 4% paraformaldehyde, and permeabilized with 0.5% Triton X-100. EdU incorporation was detected via a click chemistry reaction with the Apollo dye solution according to the manufacturer’s protocol. Nuclei were counterstained with Hoechst 33342 (RiboBio). Images were acquired using an inverted fluorescence microscope (Leica or Olympus), and the percentage of EdU-positive cells (proliferating cells) relative to the total number of Hoechst-stained cells was quantified using ImageJ software.

### 2.13 Wound-healing and Transwell invasion assays

For wound-healing (scratch) assays assessing cell migration, transfected OSCC cells (HN6, SCC25) were seeded in 6-well plates and grown to nearly 100% confluency. A linear scratch wound was created across the cell monolayer using a sterile 200 μL pipette tip. Debris was removed by washing with PBS, and cells were then incubated in low-serum medium (1% FBS) to minimize proliferation effects. Phase-contrast images were taken at 0 h, 12 h, 24 h, and 48 h. Migration was quantified in ImageJ as the distance between wound edges at each timepoint relative to 0 h. Under our culture conditions (DMEM + 10% FBS, 37°C, 5% CO_2_), the population doubling time of HN6 and SCC25 was ~33 h, which motivated the 48 h interval for migration readouts.

For Transwell invasion assays, the invasive capacity of cells was measured using Transwell chambers (8 μm pore size; Corning, NY, USA) pre-coated with Matrigel basement membrane matrix (BD Biosciences, Franklin Lakes, NJ, USA). Transfected OSCC cells (typically 2.0×10^5 cells) were resuspended in 200 μL of serum-free DMEM and seeded into the upper chamber. The lower chamber was filled with 600 μL of DMEM containing 10-20% FBS as a chemoattractant. After incubation for 24–48 hours at 37°C, non-invading cells remaining on the upper surface of the membrane were carefully removed with a cotton swab. Cells that had invaded through the Matrigel and membrane to the lower surface were fixed with 4% paraformaldehyde and stained with 0.1% crystal violet solution for 30 minutes. Invaded cells were photographed under a light microscope, and the number of cells was counted in several randomly selected fields.

### 2.14 Immune infiltration analysis

The TIMER2.0 web platform (http://timer.cistrome.org/) was utilized to systematically evaluate the correlation between GINS2 expression (log2 TPM) and the infiltration levels of various immune cell populations (CD8+ T cells, neutrophils) across TCGA cancer types, specifically focusing on the Head and Neck Squamous Cell Carcinoma (HNSC) cohort. Immune infiltration estimates were derived using multiple algorithms available on the platform (TIMER, CIBERSORT, MCP-counter). The correlation module was used to explore associations between GINS2 expression and canonical immune cell gene markers, as well as established markers of T cell exhaustion, including PD-1 (PDCD1), CTLA4, LAG3, and TIM-3 (HAVCR2). Expression scatterplots were generated, and Spearman’s rank correlation coefficient (rho) and statistical significance (p-value) were calculated and reported. Tumor purity estimates provided by the platform were also considered.

### 2.15 Isolation of CD8+ T cells and neutrophils from peripheral blood mononuclear cells

This investigation was approved by the internal ethics committee of Chinese PLA General Hospital (Approval No. S2025-018-01) and was conducted according to the principles outlined in the Declaration of Helsinki. All healthy donors provided written informed consent. Human PBMCs were isolated from buffy coats obtained from healthy donors using density gradient centrifugation over Ficoll-Paque Plus (Cytiva). Neutrophils were subsequently isolated from the granulocyte/erythrocyte pellet after Ficoll separation using dextran sedimentation followed by hypotonic lysis of remaining red blood cells, or alternatively, using immunomagnetic negative or positive selection kits targeting CD66b (Miltenyi Biotec). CD8+ T cells were isolated from the PBMC fraction using immunomagnetic negative selection kits (EasySep Human CD8+ T Cell Isolation Kit, STEMCELL Technologies) to obtain untouched CD8+ T cells. For neutrophils, cells were cultured in RPMI-1640 medium supplemented with 10% fetal bovine serum (FBS) and 1% penicillin/streptomycin. Immediately following isolation and prior to use in any experiment, neutrophil purity (>95% CD66b+) and viability (>90%) were confirmed by flow cytometry using a viability dye (e.g., 7-AAD) and Trypan blue exclusion, respectively. Only cell preparations meeting these criteria were used for subsequent functional assays. For generating tumor-reactive lymphocytes, naive CD8+ T cells could be activated using anti-CD3/CD28 beads or co-cultured with dendritic cells pulsed with tumor antigens or lysates.

All donor samples were de-identified and processed within 4 h of phlebotomy using standardized SOPs; batch IDs and operator were logged. Matched patient–neutrophil measurements were performed on the same day as tumor resection to minimize ex vivo activation.

### 2.16 Flow cytometry assay

For analysis of immune cell populations in tissues (tumors) or peripheral blood, single-cell suspensions were prepared. Tumor tissues were minced and digested enzymatically (using collagenase/DNase). Red blood cells in peripheral blood or spleen samples were lysed using ACK lysis buffer. Cells were passed through a 40-70 μm cell strainer and washed with PBS containing 2% FBS (FACS buffer). Non-specific antibody binding was blocked using Fc block (e.g., anti-mouse CD16/CD32, BD Biosciences, 553142, for mouse samples; or human Fc block for human samples) for 10–15 minutes. Cell viability was assessed using a fixable viability dye (Zombie Aqua, BioLegend; or Live/Dead Fixable Dead Cell Stain Kit, Thermo Fisher) according to the manufacturer’s instructions. For surface marker staining, cells were incubated with fluorochrome-conjugated antibodies for 30 minutes at 4°C, protected from light. Antibodies used included: anti-mouse CD11b (BioLegend, 101256, clone M1/70), anti-mouse Ly6G (BioLegend, 127628, clone 1A8), anti-human/mouse CD8, anti-human/mouse PD-1 (BioLegend, 135221, clone EH12.2H7), anti-human/mouse TIM3 (BioLegend, 345006, clone F38-2E2), anti-human CD66b, anti-human PD-L1. After staining, cells were washed twice with FACS buffer.

For detection of apoptosis, the Annexin V-FITC Apoptosis Detection Kit (BD Biosciences or similar) was used according to the manufacturer’s protocol. Briefly, cells were washed and resuspended in Annexin V binding buffer, followed by incubation with Annexin V-FITC and propidium iodide (PI) or 7-AAD for 15 minutes at room temperature in the dark. Data were acquired on a flow cytometer (BD LSRII, BD FACSCanto II, or Cytek Aurora) and analyzed using FlowJo software (v10, BD Biosciences). Appropriate gating strategies were applied based on forward scatter (FSC), side scatter (SSC), viability dye exclusion, and specific marker expression to identify and quantify populations of interest (% CD11b+Ly6G+ neutrophils, % PD1+CD8+ T cells, % apoptotic cells [Annexin V+/PI- or Annexin V+/PI+]). Mean Fluorescence Intensity (MFI) was measured for markers like PD-L1 where indicated.

**Figure 1:**
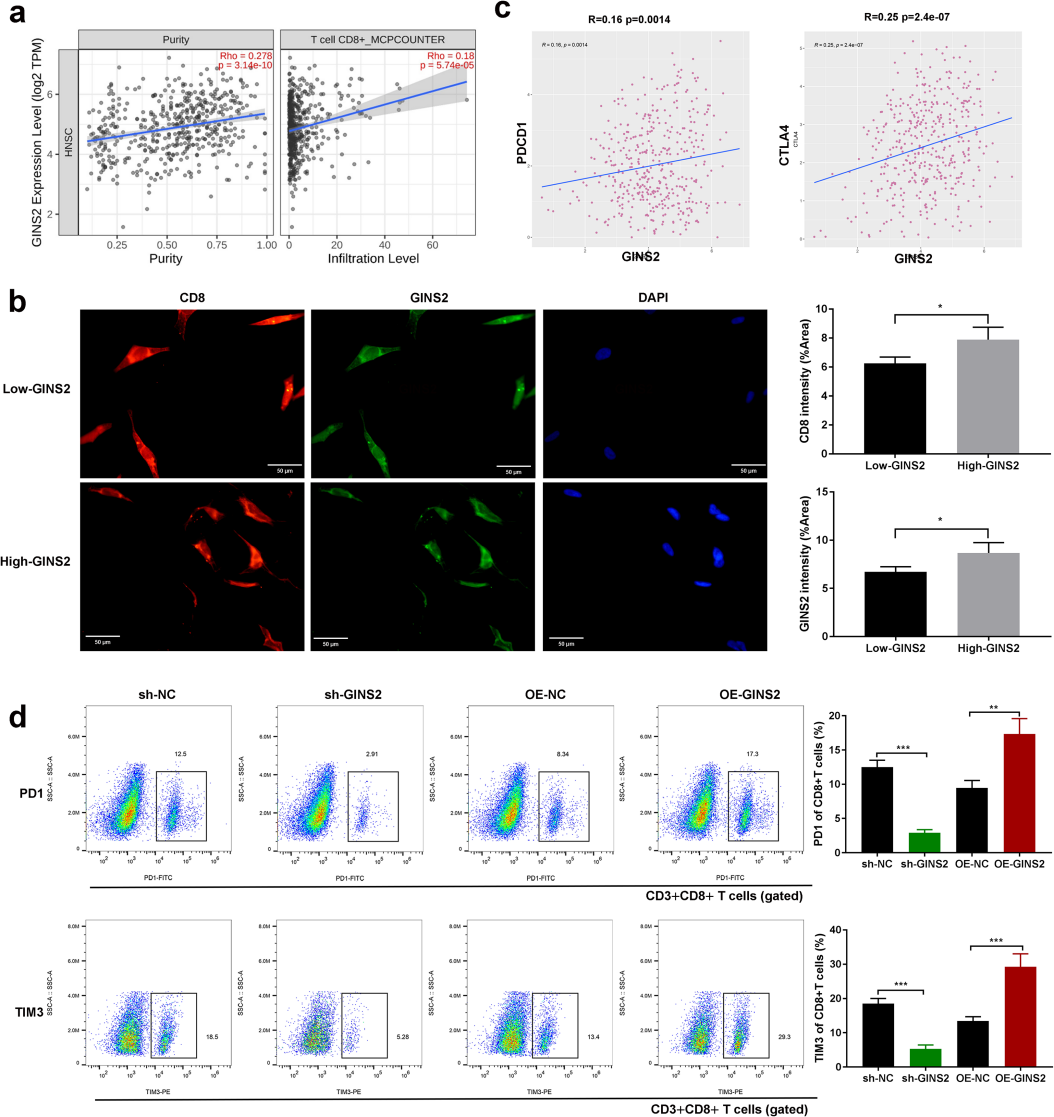
GINS2 expression correlates with T cell infiltration and exhaustion, and modulates exhaustion marker expression on CD8^+^ T cells. **(a)** Correlation analyses from TIMER2.0 (HNSC cohort). Left: scatter plot showing negative correlation between GINS2 expression (log2 TPM) and tumor purity. Right: positive correlation between GINS2 expression and CD8^+^ T cell infiltration estimated by the MCP-counter algorithm. Spearman’s rho and P-values are indicated. **(b)** Representative IF of CD8^+^ TILs (red), GINS2 (green), and DAPI (blue) in OSCC sections. CD8 marks lymphocytes, while GINS2^+^ tumor epithelial cells are CD8^-^. Clinical specimens were stratified into High-GINS2 and Low-GINS2 groups by the median H-score. Scale bar = 50 µm. **(c)** TIMER2.0 correlations in HNSC showing significant positive associations between GINS2 expression and T cell exhaustion markers PD-1 (PDCD1), LAG-3 (LAG3), and CTLA-4. Spearman’s rho and P-values are indicated. **(d)** Flow cytometry of human CD3^+^CD8^+^ T cells after co-culture with OSCC cells (sh-NC, sh-GINS2, OE-NC, OE-GINS2). Tumor cells were excluded from the CD8^+^ gate. Representative plots (left) and quantification (right) show %PD-1^+^ and %TIM-3^+^ of CD3^+^CD8^+^ T cells. Gating: live cells (FSC-A/SSC-A, viability dye^-^) → singlets (FSC-H/FSC-A) → CD3^+^CD8^+^ T cells → PD-1 and TIM-3. LAG-3 flow data were excluded due to missing batch-matched FMO/isotype controls (see Methods/Discussion). Data are presented as mean ± SD. n = 5 samples per group. *P < 0.05, **P < 0.01, ***P < 0.001.

LAG-3 staining was attempted in one acquisition batch that lacked batch-matched FMO/isotype references; consequently, LAG-3 cytometry data are not reported in the final figures. Exhaustion at the protein level is therefore shown for PD-1 and TIM-3 only; LAG3 is retained exclusively as a transcriptomic association (see **Figure 1c**). In co-culture assays, exhaustion markers (PD-1, TIM-3) were quantified on CD3^+^CD8^+^ T cells; tumor cells do not express CD3/CD8 and are excluded by these gates.

Data were acquired on BD LSR II/FACSCanto II or Cytek Aurora instruments and analyzed in FlowJo v10. Debris was excluded on FSC-A vs SSC-A, and doublets were removed using FSC-H vs FSC-A (and, when available, SSC-H vs SSC-W). Live cells were defined as Fixable Viability Dye–negative. Single-color compensation controls (cells or beads) were run each batch. Positivity thresholds for PD-1, TIM-3, PD-L1, and Annexin V/PI quadrants were set using batch-matched FMO controls; isotype controls were used for display only and not for gate placement. A minimum of 50,000 live singlet events was recorded per sample unless otherwise specified.

### 2.17 Co-immunoprecipitation analysis

Cultured OSCC cells (HN6, SCC25) were lysed in ice-cold Co-IP buffer (Tris-buffered saline pH 7.4 containing 1% NP-40 or Triton X-100, protease inhibitors, and phosphatase inhibitors). For the BC300 buffer mentioned: typically 20 mM Tris-HCl pH 8.0, 300 mM KCl, 0.2 mM EDTA, 20% glycerol, 0.1% NP-40, plus inhibitors. Lysates were cleared by centrifugation. A portion of the lysate was saved as input control. The remaining lysate was incubated overnight at 4°C with rotation with a primary antibody targeting the protein of interest (anti-GINS2, Proteintech #16247-1-AP; anti-PTP4A1, Proteintech #67584-1-Ig; or anti-PKM2, Abcam ab85555) or a control IgG antibody (normal rabbit IgG or normal mouse IgG). Protein A/G agarose beads (e.g., Santa Cruz Biotechnology or Cell Signaling Technology) were added and incubated for another 2–4 hours at 4°C to capture the antibody-protein complexes. The beads were washed extensively (3–5 times) with ice-cold lysis buffer. Immunoprecipitated proteins were eluted by boiling the beads in SDS loading buffer. Eluted samples, along with input controls, were resolved by SDS-PAGE (on a 15% gel for smaller proteins if needed, otherwise 10-12%) and analyzed by Western blotting using antibodies against the potential interaction partners (anti-PTP4A1, anti-GINS2, anti-PKM2).

### 2.18 Animal study and grouping

All animal experiments were performed in strict accordance with the guidelines approved by the Institutional Animal Care and Use Committee (IACUC) of Beijing Viewsolid Biotechnology Co. LTD and complied with the ARRIVE guidelines. Female BALB/c nude mice (for basic tumorigenicity studies) aged 4–6 weeks, or immunodeficient NOD/SCID mice (for studies involving human immune cell reconstitution) aged 5–7 weeks (18–22 g), were acquired from a certified vendor (Vital River Laboratory Animal Technology Co. Ltd, Beijing, China). Animals were housed in specific pathogen-free (SPF) controlled environments with ad libitum access to food and water, a regulated temperature (22-25°C), humidity (50-60%), and a 12-hour light/dark cycle. No specific inclusion or exclusion criteria were used beyond age, sex, and health status. Mice were acclimatized for at least one week before experiments.

Randomization was performed by an independent technician using a computer-generated list; cage and injection order were randomized to minimize confounding. Investigators measuring tumors and performing IHC scoring were blinded to group allocation until after data lock. Humane endpoints included maximum tumor diameter of 15 mm, ulceration, >15% body-weight loss, or signs of distress; no deaths occurred prior to endpoint. Exclusion criteria were prespecified (failed engraftment by day 10; injection misplacement).

*Study 1 (GINS2 knockdown effect):* BALB/c nude mice were randomly divided into 2 groups (n=5 mice per group): sh-NC and sh-GINS2. HN6 cells stably transfected with sh-NC or sh-GINS2 were harvested and resuspended in sterile PBS or Matrigel/PBS mixture. A suspension containing 2 × 10^6^ cells in 0.1-0.2 mL was injected subcutaneously into the right flank or left axillary area of each mouse. Tumor growth was monitored regularly (every 2–3 days) by measuring tumor length (L) and width (W) with calipers. Tumor volume was calculated using the formula: Volume = (L × W²)/2. Mice were monitored for health status and euthanized when tumors reached a predetermined size limit or at the study endpoint (e.g., 4 weeks post-injection). Tumors were excised, weighed, photographed, and processed for IHC or other analyses.

*Study 2 (Neutrophil depletion/Immune reconstitution/Combination therapy):* NOD/SCID mice were used. A total of 30 mice were randomly divided into five groups (n=6 mice per group): (1) PBS control, (2) T cell only, (3) T cell + Neutrophils, (4) T cell + Neutrophils + sh-GINS2 OSCC cells, (5) T cell + Neutrophils + sh-GINS2 OSCC cells + anti-PD-L1 antibody. OSCC cells (HN6-sh-GINS2 or relevant control, 5 × 10^5^ cells in 0.2 mL PBS/Matrigel) were injected subcutaneously into the flank. Human immune cells (purified T cells and neutrophils, pre-activated or co-injected at specific ratios) were administered, often intravenously or intraperitoneally, at multiple time points. Anti-human PD-L1 antibody (atezolizumab clone, appropriate dose and schedule) or control IgG was administered intraperitoneally starting when tumors became palpable. Tumor growth was monitored as described above. For neutrophil depletion studies, mice bearing OE-GINS2 tumors were treated with an anti-mouse Ly6G antibody (clone 1A8, Bio X Cell, typically 100-200 μg per mouse via IP every 2–3 days) or control IgG. At the endpoint, mice were euthanized, tumors were excised, weighed, photographed, and processed for IHC (Ki67) or flow cytometry.

### 2.19 Statistical analysis

All quantitative experimental data are presented as the mean ± standard deviation (SD) from at least three independent experiments or biological replicates, unless otherwise specified (e.g., animal studies with n=5 per group). Statistical analyses were performed using GraphPad Prism software (version 7.0; GraphPad Software, La Jolla, CA, USA). Comparisons between two groups were made using the two-tailed unpaired Student’s t-test. Comparisons among three or more groups were performed using one-way analysis of variance (ANOVA) followed by an appropriate *post-hoc* test (e.g., Tukey’s multiple comparisons test). Correlations between continuous variables (gene expression levels, MFI) were assessed using Pearson’s or Spearman’s correlation coefficient (r or rho). Survival curves (Kaplan-Meier) based on TCGA data would be compared using the log-rank (Mantel-Cox) test if applicable (though not explicitly shown in figures). A P-value less than 0.05 (P < 0.05) was considered statistically significant. Significance levels are indicated in the figures as *P < 0.05, **P < 0.01, and ***P < 0.001. Exact P-values are provided in charts or figure legends where possible.

## 3 Results

### 3.1 GINS2 is highly expressed in OSCC and correlates with aggressive clinicopathological features

**Figure 2:**
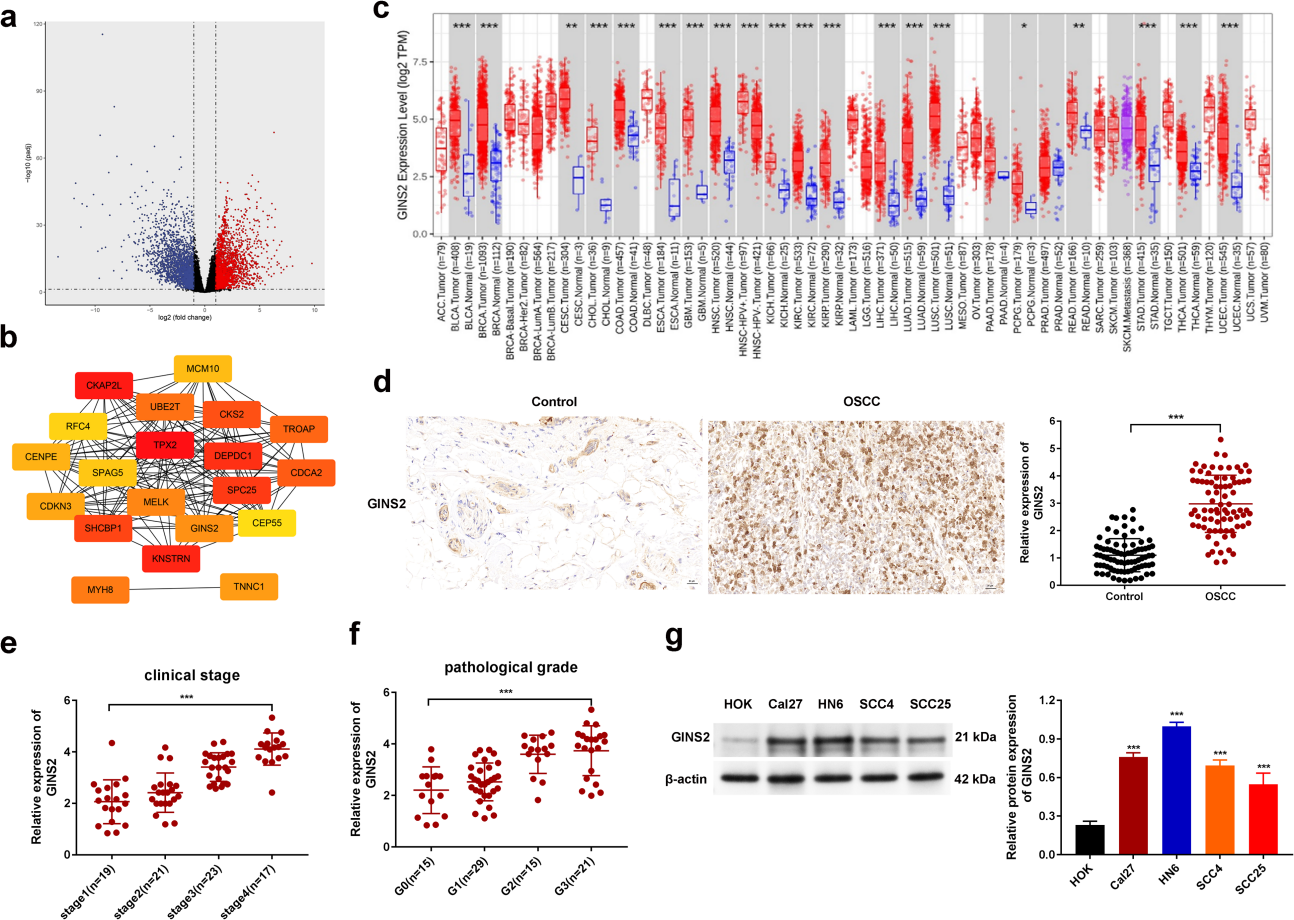
GINS2 is upregulated in OSCC and correlates with adverse clinicopathological features. **(a)** Volcano plot showing differentially expressed genes between HNSC tumor tissues and normal tissues from TCGA database. **(b)** STRING PPI network of prognostic DEGs (|log2FC| > 2; FDR < 0.05; survival-associated). Genes not present in the seed (e.g., PTP4A1, PKM) are not displayed. The panel is intended to position GINS2 within replication/cell-cycle interactors. **(c)** Pan-cancer analysis from TCGA showing GINS2 mRNA expression (log2 TPM) across cancer types (red) versus normal tissues (blue). **(d)** Representative IHC images (left) and quantification (right) of GINS2 expression in OSCC versus controls. Scale bar = 50 µm. **(e)** Relative GINS2 mRNA expression stratified by clinical stage (qPCR; OSCC patient samples). **(f)** Relative GINS2 mRNA expression stratified by pathological grade (qPCR; OSCC patient samples). **(g)** Western blot (left) and quantification (right) demonstrating GINS2 protein expression in multiple OSCC cell lines (Cal27, HN6, SCC4, SCC25) compared to normal human oral keratinocytes (HOK). β-actin served as the loading control. Data in plots are presented as mean ± SD or box plots showing median and interquartile range. *P < 0.05, **P < 0.01, ***P < 0.001.

**Figure 3:**
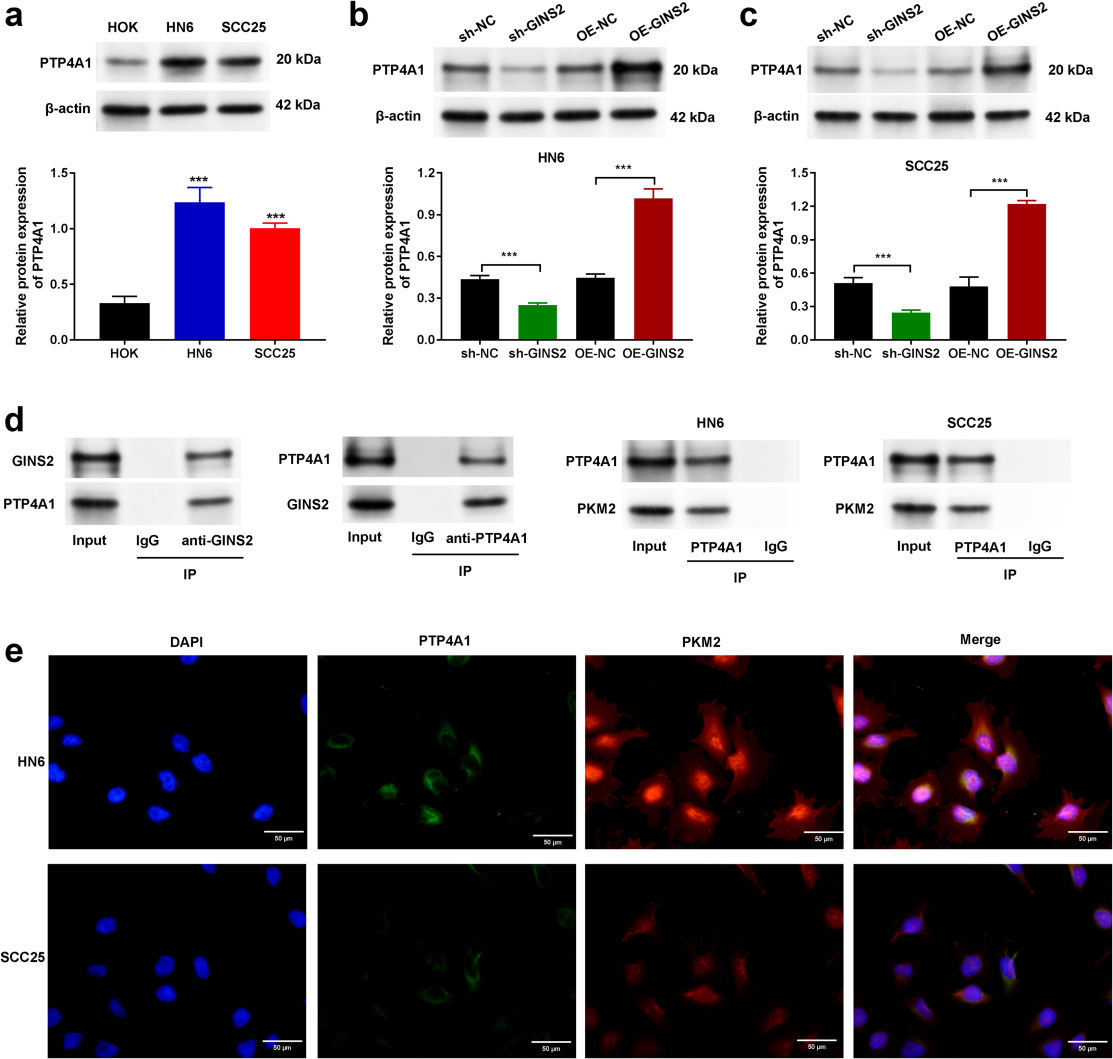
GINS2 interacts with PTP4A1 and regulates its expression, and PTP4A1 interacts with PKM2 in OSCC cells. **(a)** Western blot analysis showing baseline protein expression of PTP4A1 in normal human oral keratinocytes (HOK) and OSCC cell lines HN6 and SCC25. β-actin served as the loading control. Bar graph shows quantification relative to HOK. **(b, c)** Western blot analysis (top) and quantification (bottom) demonstrating the effect of GINS2 knockdown (sh-GINS2) or overexpression (OE-GINS2) on PTP4A1 protein levels in HN6 **(b)** and SCC25 **(c)** cells, compared to respective controls (sh-NC, OE-NC). β-actin served as the loading control. **(d)** Co-immunoprecipitation (Co-IP) assays. Left panels: Interaction between endogenous GINS2 and PTP4A1 in HN6 cells. IP with anti-GINS2 pulls down PTP4A1, and IP with anti-PTP4A1 pulls down GINS2. Right panels: Interaction between endogenous PTP4A1 and PKM2 in HN6 and SCC25 cells. IP with anti-PTP4A1 pulls down PKM2. Control IgG IPs and input lysates are shown. **(e)** Immunofluorescence staining showing co-localization of PTP4A1 (green) and PKM2 (red) in HN6 and SCC25 cells. Nuclei are stained with DAPI (blue). Scale bar = 20 µm. Data in bar graphs are presented as mean ± SD. ***P < 0.001.

To investigate the potential role of GINS2 in OSCC pathogenesis, we first performed bioinformatic analyses using TCGA data. Differential expression analysis revealed GINS2 as one of the significantly upregulated genes in HNSC tissues compared to normal tissues (**Figure 2a**). A PPI network constructed using prognostic differentially expressed genes highlighted GINS2 as a potential hub node, suggesting its central role in relevant biological pathways (**Figure 2b**). **Figure 2b** is therefore a seed-constrained network positioning GINS2 within replication/cell-cycle neighborhoods, consistent with its CMG-helicase function. PTP4A1 and PKM (PKM2 isoform) are absent because they did not satisfy the seed criteria in this dataset; they are introduced mechanistically in **Figure 3** based on co-immunoprecipitation and co-localization data. Pan-cancer analysis across multiple TCGA tumor types confirmed that GINS2 expression is significantly elevated in numerous malignancies, including HNSC, compared to corresponding normal tissues (**Figure 2c**). We then validated these findings in clinical OSCC samples. IHC staining demonstrated significantly higher GINS2 protein expression in OSCC tumor tissues compared to adjacent non-cancerous control tissues (**Figure 2d**). Analysis of GINS2 expression relative to clinicopathological parameters revealed a positive correlation with advanced clinical stage (Stage I vs. II vs. III) (**Figure 2e**) and higher pathological tumor grade (G1 vs. G2 vs. G3) (**Figure 2f**). Consistent with the tissue data, Western blot analysis showed markedly higher GINS2 protein levels in various human OSCC cell lines (Cal27, HN6, SCC4, SCC25) compared to normal human oral keratinocytes (HOK) (**Figure 2g****).**

Collectively, these results establish that GINS2 is significantly overexpressed in OSCC, and its high expression is associated with more advanced and aggressive disease characteristics.

### 3.2 GINS2 promotes OSCC proliferation, migration, invasion, and tumorigenesis

**Figure 4:**
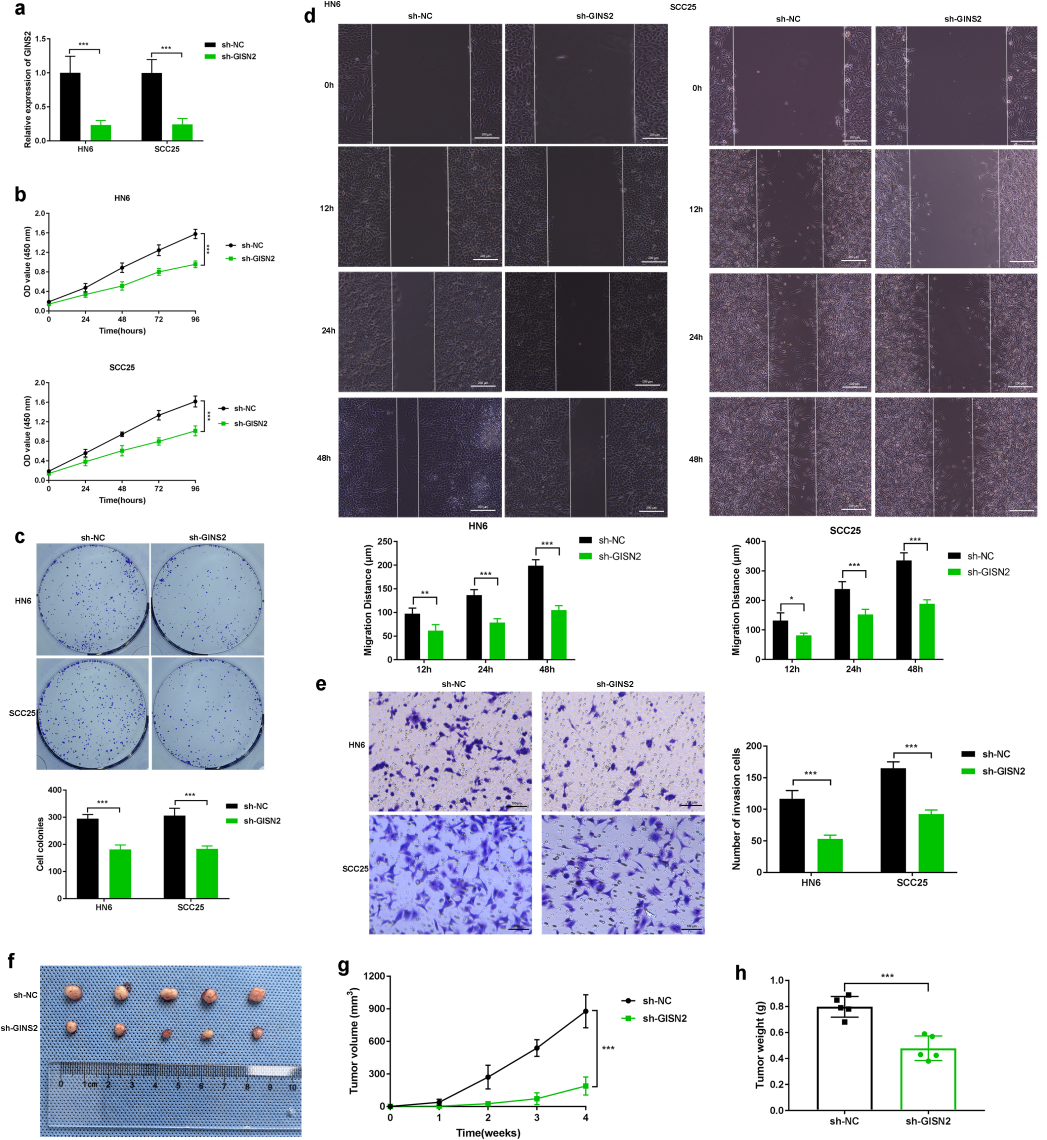
GINS2 promotes OSCC proliferation, migration, invasion, and tumorigenesis. **(a)** qPCR confirming efficient knockdown of GINS2 mRNA in HN6 and SCC25 cells after sh-GINS2 transfection compared to sh-NC. β-actin was used for normalization. **(b)** CCK-8 assay over 96 hours showing reduced viability of HN6 and SCC25 cells upon GINS2 knockdown. **(c)** Colony formation assay images (top) and quantification (bottom) demonstrating fewer and smaller colonies after GINS2 silencing. **(d)** Wound-healing assay images (0 h, 12 h, 24 h, and 48 h) and quantification showing impaired migration. Scale bar = 200 µm. **(e)** Transwell Matrigel invasion assay images (left) and quantification (right) showing reduced invasion after GINS2 silencing. Invaded cells stained with crystal violet. Scale bar = 100 µm. **(f–h)** Effect of GINS2 knockdown on OSCC tumor growth *in vivo*. Representative xenograft images **(f)**, tumor growth curves **(g)**, and final tumor weight **(h)** showing suppressed tumorigenesis in sh-GINS2 vs sh-NC HN6 cells. Data are presented as mean ± SD. ***P < 0.001. n = 5 mice per group.

Given the elevated expression of GINS2 in OSCC, we next sought to determine its functional role in OSCC progression using loss-of-function approaches in HN6 and SCC25 cell lines. To delineate tumor cell–intrinsic functions, all *in vitro* assays in this subsection were performed without immune cells, and xenografts were established in nude mice without adoptive immune reconstitution. We efficiently knocked down GINS2 expression using specific shRNA (sh-GINS2), confirmed by qPCR analysis showing significantly reduced GINS2 mRNA levels compared to control cells transfected with non-targeting shRNA (sh-NC) (**Figure 4a**). Functionally, GINS2 knockdown significantly inhibited cell viability and proliferation over time, as assessed by CCK-8 assays (**Figure 4b**). The ability of OSCC cells to form colonies was markedly reduced upon GINS2 silencing (**Figure 4c**). Plating efficiency and baseline surviving fractions for HN6 and SCC25 are provided in **Supplementary Figure S1**. We also examined the impact of GINS2 on cell motility. Wound-healing assays revealed that GINS2 knockdown significantly impaired the migratory capacity of both HN6 and SCC25 cells, as evidenced by reduced migration distance at 12 h, 24 h, and 48 h compared with controls (**Figure 4d**). Similarly, Transwell Matrigel invasion assays demonstrated that silencing GINS2 significantly reduced the invasive potential of OSCC cells (**Figure 4e**). To validate these findings *in vivo*, HN6 cells stably expressing sh-NC or sh-GINS2 were subcutaneously injected into nude mice. Consistent with the *in vitro* results, GINS2 knockdown led to a significant suppression of xenograft tumor growth, resulting in substantially smaller tumor volumes and lower tumor weights compared to the control group after 4 weeks (**Figure 4f**).

Taken together, these data indicate a cell-intrinsic role for GINS2 in promoting proliferation, clonogenic growth, migration, invasion, and baseline xenograft expansion *in vivo.*

### 3.3 GINS2 expression correlates with immune cell infiltration and T cell exhaustion markers

The analyses in this subsection interrogate tumor microenvironmental/immune correlates rather than cell-autonomous tumor properties. Recognizing the crucial role of the TME in cancer progression, we investigated the relationship between GINS2 expression and immune infiltration in OSCC using TCGA data analyzed via TIMER2.0. GINS2 expression showed significant positive correlation with estimated tumor purity (Rho=0.278, **Figure 1a**, left panel), and also exhibited a significant negative correlation with CD8+ T cell infiltration levels estimated by the MCP-counter algorithm (Rho=0.18, **Figure 1a**, right panel). Consistent with a broader cytotoxic compartment, GINS2 also showed a weak but significant correlation with NK-cell infiltration estimated by MCP-counter (ρ = 0.126, p = 5.14×10^-^³; **Supplementary Figure S2**). However, further analysis revealed significant positive correlations between GINS2 expression levels (log2 TPM) and the expression of key T cell exhaustion markers, including PDCD1 (encoding PD-1), LAG3, and CTLA4, in the HNSC cohort (**Figure 1c**). Immunofluorescence of patient OSCC sections identified CD8^+^ tumor-infiltrating lymphocytes (TILs); epithelial tumor cells (GINS2^+^) showed no CD8 signal. High-GINS2 cases displayed higher CD8^+^ TIL signal than Low-GINS2 cases (**Figure 1b**), suggesting potential regulation or co-enrichment. To functionally link GINS2 to T cell exhaustion phenotype, we performed flow cytometry on antigen-specific CD8+ T cells co-cultured with OSCC (HN6/SCC25) cells manipulated for GINS2 expression. Flow cytometry readouts are from CD3^+^CD8^+^ T cells gated within the lymphocyte compartment; OSCC cells lack CD3/CD8 and are excluded by lineage and scatter gates. Knockdown of GINS2 (sh-GINS2) led to a significant decrease in the percentage of CD8+ T cells expressing the exhaustion markers PD-1 and TIM3 compared to co-culture with control (sh-NC) OSCC cells. Conversely, overexpression of GINS2 (OE-GINS2) significantly increased the percentage of CD8+ T cells positive for PD-1 and TIM3 (**Figure 1d**). Immunofluorescence on patient tumors showed that High-GINS2 cases (H-score ≥ median) displayed higher CD8 signal than Low-GINS2 cases (H-score < median) (**Figure 1b**).

Thus, the PD-1/TIM-3 modulation observed here reflects microenvironment-dependent effects that arise during tumor–T-cell interactions.

### 3.4 GINS2 promotes neutrophil infiltration, and TANs contribute to GINS2-mediated tumor growth

**Figure 5:**
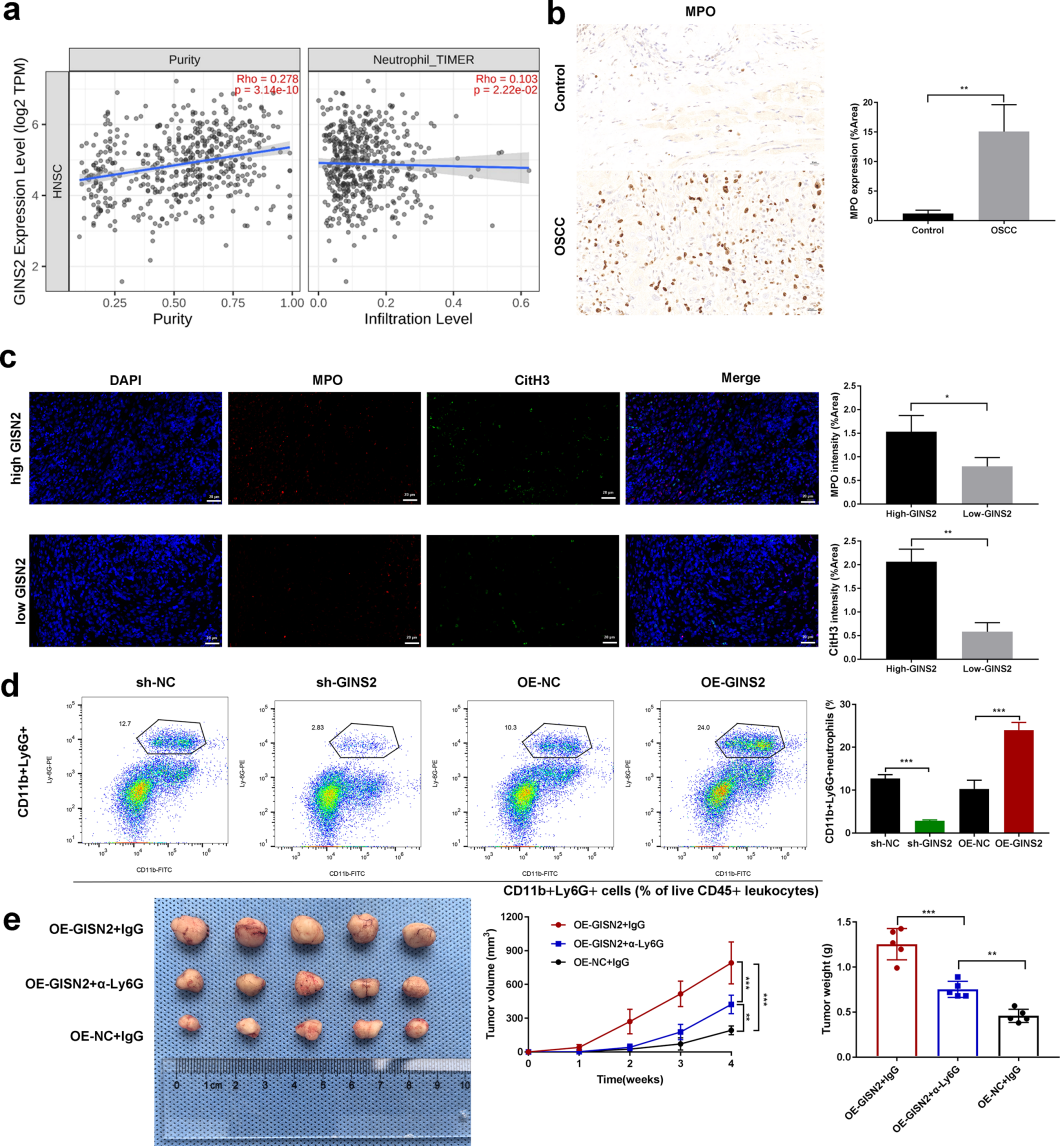
GINS2 expression correlates with neutrophil infiltration, GINS2 promotes TAN accumulation, and neutrophil depletion impairs GINS2-driven tumor growth. **(a)** Correlation analyses using TIMER2.0 database in the HNSC cohort. Left: Scatter plot showing correlation between GINS2 expression level (log2 TPM) and estimated tumor purity. Right: Scatter plot showing correlation between GINS2 expression level and neutrophil infiltration level estimated by the TIMER algorithm. Spearman’s rho and P-value are indicated. **(b)** Left: Representative immunohistochemistry (IHC) staining for Myeloperoxidase (MPO) in control oral tissue and OSCC tissue, showing increased neutrophil infiltration in OSCC. Right: Quantification of MPO expression (% Area) from IHC images (n=5 per group). Scale bar = 50 µm. **(c)** Left: Representative immunofluorescence images showing staining for MPO (red) and citrullinated histone H3 (CitH3, green) in OSCC tissue sections. Nuclei are stained with DAPI (blue). Merged images suggest potential NET formation. Right: corresponding quantification. Scale bar = 50 µm. **(d)** Flow cytometry analysis quantifying the percentage of CD11b+Ly6G+ neutrophils within subcutaneous xenograft tumors derived from HN6 cells transfected with sh-NC, sh-GINS2, OE-NC, or OE-GINS2. Gating strategy: live (viability dye^-^) → CD45^+^ leukocytes → CD11b^+^Ly6G^+^ neutrophils. Readout: %CD11b^+^Ly6G^+^ of CD45^+^ cells within the tumor digest. Representative flow plots (left) and quantification (right) are shown. **(e)** Effect of neutrophil depletion on GINS2-driven tumor growth *in vivo*. Representative images of subcutaneous xenograft tumors derived from OE-GINS2 HN6 cells in nude mice treated with control IgG or anti-Ly6G antibody (left), and quantification of tumor volume (middle) over time and final tumor weight (right). Data are presented as mean ± SD. **P < 0.01, ***P < 0.001.

Here we examine microenvironmental consequences of tumor-cell GINS2—specifically, neutrophil (TAN) recruitment. So, we investigated the association between GINS2 and neutrophil infiltration. TCGA analysis using TIMER2.0 showed a positive correlation between GINS2 expression level and estimated neutrophil infiltration in the HNSC cohort (**Figure 5a**, right panel; correlation with purity shown left). Consistent with neutrophil presence in OSCC, IHC staining confirmed abundant infiltration of MPO-positive neutrophils within human OSCC tumor tissues compared to minimal presence in control tissues (**Figure 5b**). Immunofluorescence staining suggested potential co-localization or proximity of MPO-positive neutrophils and cells expressing citrullinated histone H3 (CitH3), a marker often associated with neutrophil extracellular traps (NETs), within the tumor milieu (**Figure 5c**). To functionally assess the impact of GINS2 on neutrophil recruitment *in vivo*, we analyzed neutrophil populations in xenograft tumors derived from OSCC cells engineered to overexpress GINS2 (OE-GINS2) or to express a GINS2-targeting shRNA (sh-GINS2), alongside their matched controls (OE-NC, sh-NC). Flow cytometry analysis revealed that tumors derived from OE-GINS2 HN6 cells contained a significantly higher percentage of CD11b+Ly6G+ neutrophils compared to control (OE-NC) tumors. Conversely, tumors from GINS2-knockdown (sh-GINS2) cells exhibited a significantly lower percentage of these neutrophils compared to sh-NC tumors (**Figure 5d**). To determine if these recruited neutrophils contribute to GINS2-driven tumor progression, we performed a neutrophil depletion study. Mice bearing OE-GINS2 HN6 xenografts were treated with an anti-Ly6G antibody to deplete neutrophils or with control IgG. Neutrophil depletion significantly suppressed the enhanced tumor growth observed in the OE-GINS2 group, reducing both tumor volume and weight compared to IgG-treated OE-GINS2 controls (**Figure 5e**).

Because neutrophil depletion abrogated the OE-GINS2 growth advantage, these *in vivo* effects are attributable to microenvironmental TANs rather than purely cell-intrinsic proliferation.

### 3.5 GINS2 interacts with PTP4A1 and regulates its expression, while PTP4A1 interacts with PKM2

All mechanistic experiments in this section were conducted in tumor cells (cell-intrinsic context). To explore the molecular mechanisms underlying GINS2’s functions, we investigated potential interacting partners and downstream effectors. Following, investigation of the baseline expression of PTP4A1 in HOK, HN6, and SCC25 cells (**Figure 3a**) we examined whether GINS2 affects PTP4A1 expression. Western blot analysis showed that GINS2 knockdown (sh-GINS2) led to a decrease in PTP4A1 protein levels, while GINS2 overexpression (OE-GINS2) resulted in increased PTP4A1 protein levels in both HN6 (**Figure 3b**) and SCC25 cells (**Figure 3c**), compared to their respective controls (sh-NC, OE-NC). Next, we performed Co-IP assays to test for direct interactions. Immunoprecipitation of endogenous GINS2 successfully pulled down PTP4A1, and conversely, immunoprecipitation of endogenous PTP4A1 co-precipitated GINS2 in HN6 cell lysates, suggesting a physical interaction between GINS2 and PTP4A1 (**Figure 3d**, left panels). Given PTP4A1’s known roles and potential links to metabolic enzymes like PKM2, we also investigated the interaction between PTP4A1 and PKM2. Co-IP assays demonstrated that immunoprecipitation of endogenous PTP4A1 resulted in the co-precipitation of PKM2 in both HN6 and SCC25 cell lysates (**Figure 3d**, right panels). To visualize the potential proximity of PTP4A1 and PKM2 within cells, we performed immunofluorescence staining. The results showed significant co-localization of PTP4A1 and PKM2 signals, particularly in the cytoplasm and potentially near the cell periphery, in both HN6 and SCC25 cells (**Figure 3e**).

These findings suggest that GINS2 may exert its effects, at least in part, by interacting with and potentially regulating PTP4A1, which itself interacts and co-localizes with the key metabolic enzyme PKM2 in OSCC cells. Because we did not assay PTP4A1 transcripts in these experiments, we conservatively interpret the GINS2-dependent changes as effects on steady-state protein abundance. Together with the reciprocal Co-IP, these data support a model in which GINS2 directly engages PTP4A1 and increases its availability to form a complex with PKM2.

### 3.6 GINS2-associated neutrophils express PD-L1 and mediate immunosuppression; targeting GINS2 synergizes with anti-PD-L1 therapy

**Figure 6:**
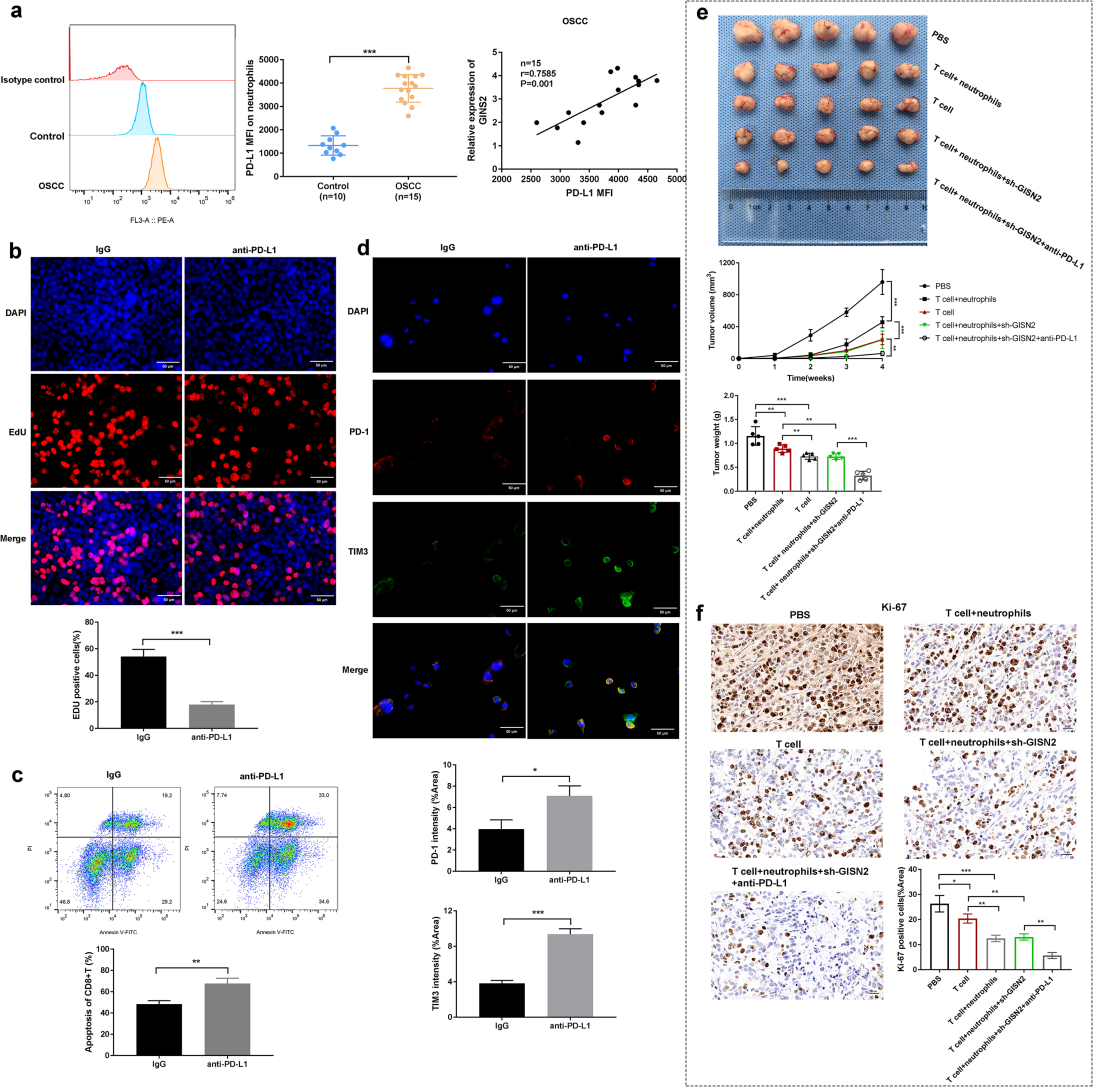
GINS2-associated neutrophils express PD-L1, mediating immunosuppression, and targeting GINS2 synergizes with anti-PD-L1 therapy *in vivo*. **(a)** Left: Representative flow cytometry histogram showing higher PD-L1 surface expression (MFI) on neutrophils from OSCC environment compared to control. **(a)** Right: Dot plot showing a significant positive correlation between GINS2 relative expression (in matched tumors) and PD-L1 MFI on associated neutrophils (n = 15 matched OSCC cases, Spearman correlation). Gating strategy: live (viability dye^-^) → CD66b^+^ neutrophils → PD-L1 (PE-conjugated) histogram/overlay. Readouts: PD-L1 MFI and %PD-L1^+^ among CD66b^+^ cells (FMO-anchored). **(b)** EdU incorporation assay assessing proliferation of CD8+ T cells co-cultured with neutrophils +/- anti-PD-L1 antibody or control IgG. Representative images (upper) and quantification (lower) show increased T cell proliferation upon PD-L1 blockade. Scale bar = 50 µm. **(c)** Flow cytometry analysis assessing apoptosis (Annexin V-FITC/PI staining) of CD8+ T cells after co-culture and treatment with control IgG or anti-PD-L1 antibody. Upper: Representative flow plots. Lower: Quantification of the percentage of apoptotic (Annexin V+) CD8+ T cells. Gating strategy: lymphocytes (FSC-A vs SSC-A) → singlets (FSC-H/FSC-A) → live (viability dye^-^) → CD3^+^CD8^+^ T cells → Annexin V-FITC vs PI. Readouts: %Annexin V^+^ (early + late apoptosis) among CD3^+^CD8^+^ cells; PI used to distinguish early (Annexin V^+^/PI^-^) vs late (Annexin V^+^/PI^+^) apoptosis when reported. **(d)** Representative immunofluorescence images showing expression of PD-1 (red) and TIM-3 (green) on cells (likely T cells) within the tumor microenvironment. Nuclei are stained with DAPI (blue). Scale bar = 50 µm. **(e)***In vivo* therapeutic efficacy study using NOD/SCID mice reconstituted with human T cells and neutrophils, injected with sh-GINS2 HN6 cells, and treated with PBS, control IgG, or anti-PD-L1 antibody. Representative images of excised tumors (upper), and quantification of tumor volume over time (middle) and final tumor weight (lower) across different groups: PBS, T cell only, T cell+neutrophils, T cell+neutrophils+sh-GINS2, T cell+neutrophils+sh-GINS2+anti-PD-L1. **(f)** Representative IHC staining for Ki67 in xenograft tumors from the different treatment groups in the *in vivo* study described in **(d)**. Scale bar = 50 µm. Data are presented as mean ± SD. *P < 0.05, **P < 0.01, ***P < 0.001.

All assays in this subsection require immune components and therefore test microenvironment-mediated effects. Given that TANs can suppress T cell function via PD-L1, we examined PD-L1 expression on neutrophils in the OSCC context. Flow cytometry analysis of neutrophils isolated from OSCC patient samples or potentially tumor xenografts revealed significantly higher surface PD-L1 MFI compared to neutrophils from healthy controls (**Figure 6a**, left panels). Importantly, we found a significant positive correlation between GINS2 mRNA expression levels (potentially from bulk tumor or sorted tumor cells) and the PD-L1 MFI on associated neutrophils (r=0.7885, P<0.001; n = 15 matched OSCC cases; **Figure 6a**, right panel), suggesting GINS2 expression levels influence the immunosuppressive phenotype of TANs. To test the functional consequence of PD-L1 expression on TANs, we performed *in vitro* assays. Co-culture experiments involving T cells and neutrophils demonstrated-surprisingly-that blocking PD-L1 using an anti-PD-L1 antibody significantly decreased CD8+ T cell proliferation (potentially indicating an unexpected effect or specific context in this assay) as measured by EdU incorporation (**Figure 6b**). We also observed increased apoptosis (Annexin V positivity) of CD8+ T cells upon anti-PD-L1 treatment compared to IgG control (**Figure 6c**). Immunofluorescence staining in tumor sections confirmed the presence of T cells expressing exhaustion markers PD-1 and TIM3, with increased intensity observed after anti-PD-L1 treatment compared to IgG (**Figure 6d**). Finally, we evaluated the therapeutic potential of combining GINS2 targeting with PD-L1 blockade in an *in vivo* immune reconstitution model using NOD/SCID mice bearing HN6 xenografts. Mice were inoculated with sh-GINS2 OSCC cells along with human T cells and neutrophils. Compared to controls (PBS, T cells only, T cells + neutrophils), the combination of T cells + neutrophils + sh-GINS2 cells showed reduced tumor growth. Critically, the addition of anti-PD-L1 antibody to the T cell + neutrophil + sh-GINS2 group resulted in the most significant suppression of tumor volume and weight (**Figure 6e**). IHC analysis of these tumors revealed the lowest Ki67 proliferation index in the combination therapy group (T cell + neutrophil + sh-GINS2 + anti-PD-L1), compared to groups receiving T cells + neutrophils or T cells alone (**Figure 6f**).

Together with Section 3.2, these findings separate GINS2’s cell-intrinsic growth programs (**Figure 4**) from microenvironmental immunosuppression mediated by TAN PD-L1 and T-cell dysfunction (**Figures 5**, **6**).

## 4 Discussion

OSCC remains a formidable clinical challenge, driven by complex molecular alterations and intricate interactions within the tumor microenvironment (31). Our study identifies GINS2, a core component of the DNA replication machinery, as a significant oncogenic driver and a key modulator of the immune landscape in OSCC. We provide comprehensive evidence demonstrating that GINS2 is markedly overexpressed in OSCC tissues and cell lines, where its high levels correlate strongly with advanced clinical stage and pathological grade, indicating its potential as a prognostic biomarker. This aligns with findings in other cancer types where GINS2 upregulation is frequently associated with aggressive behavior and poor outcomes (24, 32, 33), reinforcing the idea that dysregulation of fundamental DNA replication components is a common feature contributing to malignancy.

Functionally, our *in vitro* and *in vivo* experiments unequivocally establish GINS2’s role in promoting core cancer hallmarks in OSCC. Silencing GINS2 robustly inhibited OSCC cell proliferation, colony formation, migration, and invasion, while also significantly curtailing xenograft tumor growth in nude mice. These findings are consistent with GINS2’s canonical function in facilitating DNA replication, providing the necessary machinery for rapid cell division characteristic of cancer (34). Beyond proliferation, the observed impact on migration and invasion suggests GINS2 might possess non-canonical functions or influence pathways regulating cell motility, contributing to the metastatic potential of OSCC.

Seeking mechanistic insights, we uncovered a proximal GINS2–PTP4A1–PKM2 module. Reciprocal endogenous Co-IP demonstrated that GINS2 physically associates with PTP4A1, and bidirectional manipulation of GINS2 levels monotonically altered PTP4A1 steady-state protein abundance, consistent with a protein-level mechanism (e.g., stabilization and/or scaffolding) rather than primary transcriptional control. PTP4A1 is an oncogenic phosphatase that promotes metastasis and proliferation (27). We further showed that PTP4A1 interacts and co-localizes with PKM2, a pivotal glycolytic enzyme that also executes non-metabolic signaling functions in cancer (35, 36). In this study we did not assay downstream signaling (PI3K/AKT, MAPK) or PKM2-dependent glycolytic flux and therefore refrain from pathway-level claims. Nevertheless, the observed binding relationships and GINS2-dependent modulation of PTP4A1 abundance support a working model in which GINS2 increases PTP4A1 availability to assemble with PKM2, potentially linking replication machinery to signaling and metabolic reprogramming in OSCC (25, 28).

A particularly significant finding of our study is the profound impact of GINS2 on the OSCC immune microenvironment. While TCGA analysis showed a complex picture regarding overall CD8+ T cell infiltration, it clearly revealed a strong positive correlation between GINS2 expression and multiple T cell exhaustion markers (PDCD1, LAG3, CTLA4). This was functionally corroborated by our *in vitro* co-culture experiments, where OSCC cell GINS2 levels modulated PD-1 and TIM-3 on interacting CD8^+^ T cells (with LAG3 support remaining transcriptomic in TCGA and not analyzed by cytometry due to quality-control constraints). Taken together with our *in vivo* mediator experiments (Ly6G neutrophil depletion and PD-L1 blockade), we interpret the T-cell phenotype as indirect—arising via PD-L1^+^ tumor-associated neutrophils—rather than direct transcriptional control by GINS2.

Furthermore, our study highlights a critical role for GINS2 in orchestrating neutrophil infiltration and function within the OSCC TME. We observed a positive correlation between GINS2 expression and neutrophil infiltration signatures in TCGA data, which was validated *in vivo* where GINS2 overexpression increased TAN accumulation, while GINS2 knockdown reduced it. Crucially, depleting these neutrophils using an anti-Ly6G antibody significantly attenuated the tumor-promoting effect of GINS2 overexpression, demonstrating that TANs are key mediators of GINS2’s oncogenic function. This aligns with the growing body of evidence supporting a pro-tumorigenic role for TANs in many cancers, including OSCC (30, 37). The mechanisms by which GINS2 promotes neutrophil recruitment warrant further investigation; it may involve the upregulation of neutrophil-attracting chemokines (e.g., CXCL1, CXCL2, CXCL8) either directly or indirectly via pathways like PTP4A1/PKM2 signaling (38).

Our investigation delved deeper into the functional phenotype of these GINS2-associated TANs, revealing elevated PD-L1 expression on neutrophils from OSCC environments compared to controls. The strong positive correlation between tumor GINS2 levels and neutrophil PD-L1 MFI suggests that GINS2 not only recruits neutrophils but also shapes their immunosuppressive potential. Neutrophil PD-L1 expression is increasingly recognized as a significant mechanism of T cell suppression in cancer (23, 39). Our functional assays confirmed this, showing that anti-PD-L1 treatment could reverse T cell proliferation and potentially viability in co-cultures containing neutrophils. This interplay culminated in our *in vivo* therapeutic experiments using an immune-reconstituted mouse model. Targeting GINS2 via shRNA knockdown combined with T cells and neutrophils showed anti-tumor activity, but the addition of anti-PD-L1 blockade yielded the most profound tumor suppression, accompanied by reduced Ki67 expression. This provides compelling preclinical evidence that the GINS2 pathway fosters immune evasion largely through PD-L1+ neutrophils, and that combining GINS2 pathway inhibition with PD-L1/PD-1 checkpoint blockade could be a synergistic therapeutic strategy for OSCC.

Our *in-vivo* approach intentionally employed a reductionist human OSCC xenograft configuration with adoptive human T cells and neutrophils to isolate the mechanistic sequence whereby tumor-intrinsic GINS2 promotes TAN recruitment, induces PD-L1 on TANs, and suppresses CD8^+^ T-cell function. This design avoids cross-species confounds inherent to murine syngeneic models and circumvents the known variability and cytokine-driven biases of myeloid compartments in HSC-reconstituted humanized strains, which can alter neutrophil checkpoint phenotypes. Consistent results across human datasets, patient tissues, ex vivo human co-cultures, and *in-vivo* necessity/PD-L1-dependence tests support the causal, human-relevant GINS2→TAN→PD-L1 axis we report. We note that carcinogen-induced or gene-edited syngeneic OSCC and next-generation humanized platforms will be valuable to extend these findings to broader immune ecosystems and long-term co-evolution, but such models address generalizability, not the validity of the mechanistic link established here.

Beyond PD-L1–mediated suppression, recent work shows that neutrophils can actively drive immunotherapy resistance by recruiting immunosuppressive CCR5^+^ T cells. In a bispecific anti-TGF-β/PD-L1 setting (YM101), neutrophil activation increased CCL3/CCL4 expression and promoted CCR5^+^ T-cell accumulation; depleting neutrophils reduced CCR5^+^ T cells and abolished the therapeutic synergy from adding the CCR5 antagonist maraviroc, whereas CCR5 blockade restored response by preventing this feedback loop (40). These data position our finding of PD-L1^+^ TAN recruitment in OSCC within a broader paradigm of neutrophil-driven immune evasion and resistance, and motivate testing chemokine-axis interventions (e.g., CCR5 blockade) alongside PD-L1 inhibition in OSCC models where TANs are prominent.

Despite the robustness of our findings, certain limitations exist. The study relies partly on correlations from database analyses and *in vitro* assays. While the *in vivo* xenograft models, including the immune reconstitution experiments, provide stronger evidence, they still may not fully recapitulate the complexity of the human OSCC TME and immune system dynamics. Future studies employing immunocompetent syngeneic OSCC models or patient-derived organoid co-culture systems would be valuable for validation. The precise molecular mechanisms linking GINS2 to PTP4A1 regulation, neutrophil chemokine production, and PD-L1 upregulation on neutrophils remain to be fully elucidated. Investigating direct transcriptional targets of GINS2 (potentially through its interaction with transcription factors) or signaling pathways downstream of the GINS2/PTP4A1/PKM2 axis could provide these missing links.

In conclusion, our study delineates a critical oncogenic role for GINS2 in OSCC, extending beyond its canonical function in DNA replication. We demonstrate that GINS2 promotes tumor cell proliferation, migration, and invasion, potentially involving interaction with PTP4A1 and PKM2. Significantly, GINS2 actively shapes an immunosuppressive tumor microenvironment by correlating with T cell exhaustion markers and, crucially, by promoting the recruitment of pro-tumorigenic neutrophils that express high levels of PD-L1, thereby inhibiting anti-tumor T cell responses. Our findings strongly suggest that the GINS2 pathway represents a promising therapeutic target in OSCC, and that strategies combining GINS2 inhibition with PD-L1/PD-1 axis blockade hold significant potential for overcoming immune evasion and improving treatment outcomes for patients with this challenging malignancy.

## Data Availability

The original contributions presented in the study are included in the article/**Supplementary Files**, further inquiries can be directed to the corresponding author/s.
