# Supplementary material for: GINS2 promotes oral squamous cell carcinoma progression and immune evasion by recruiting PD-L1+ neutrophils and modulating the PTP4A1/PKM2 axis
Source: Front Immunol. 2025 Nov 5;16:1637296. doi: 10.3389/fimmu.2025.1637296 (PMC12658745; doi:10.3389/fimmu.2025.1637296)
Supplement: Supplementary file 1 [file Table1.docx]

**Supplementary Figures**


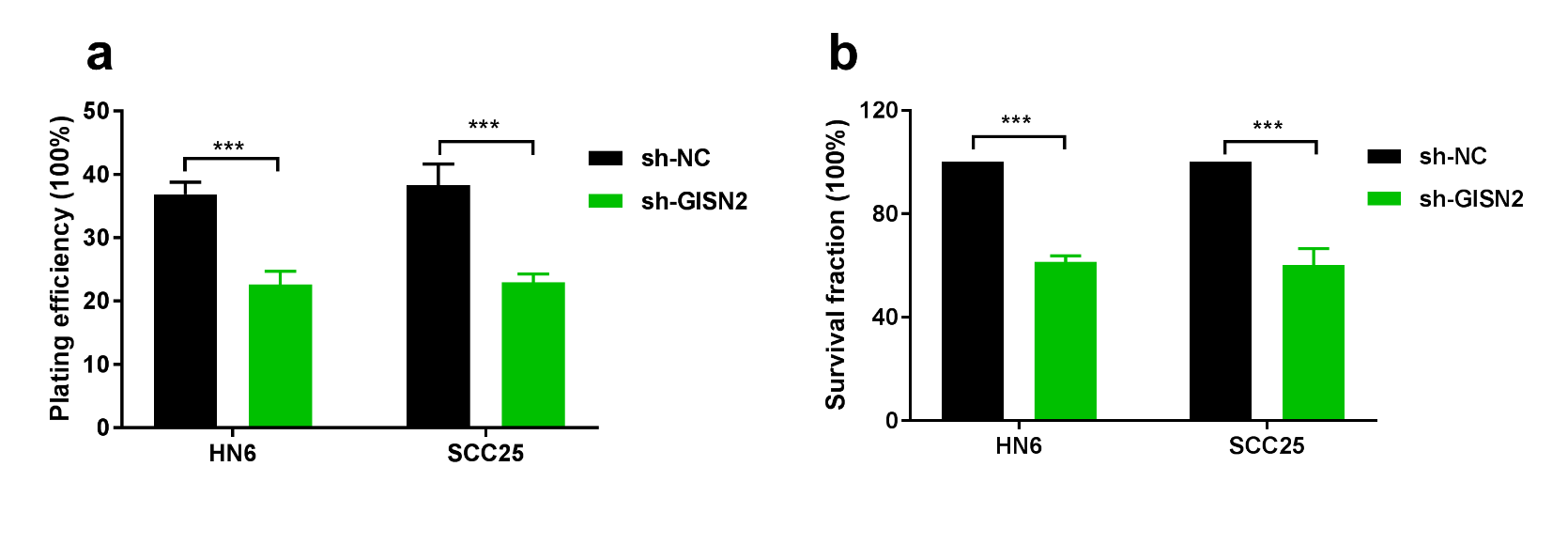


**Supplementary Figure S1.** Plating efficiency and surviving fraction in colony-formation assays for HN6 and SCC25.

**
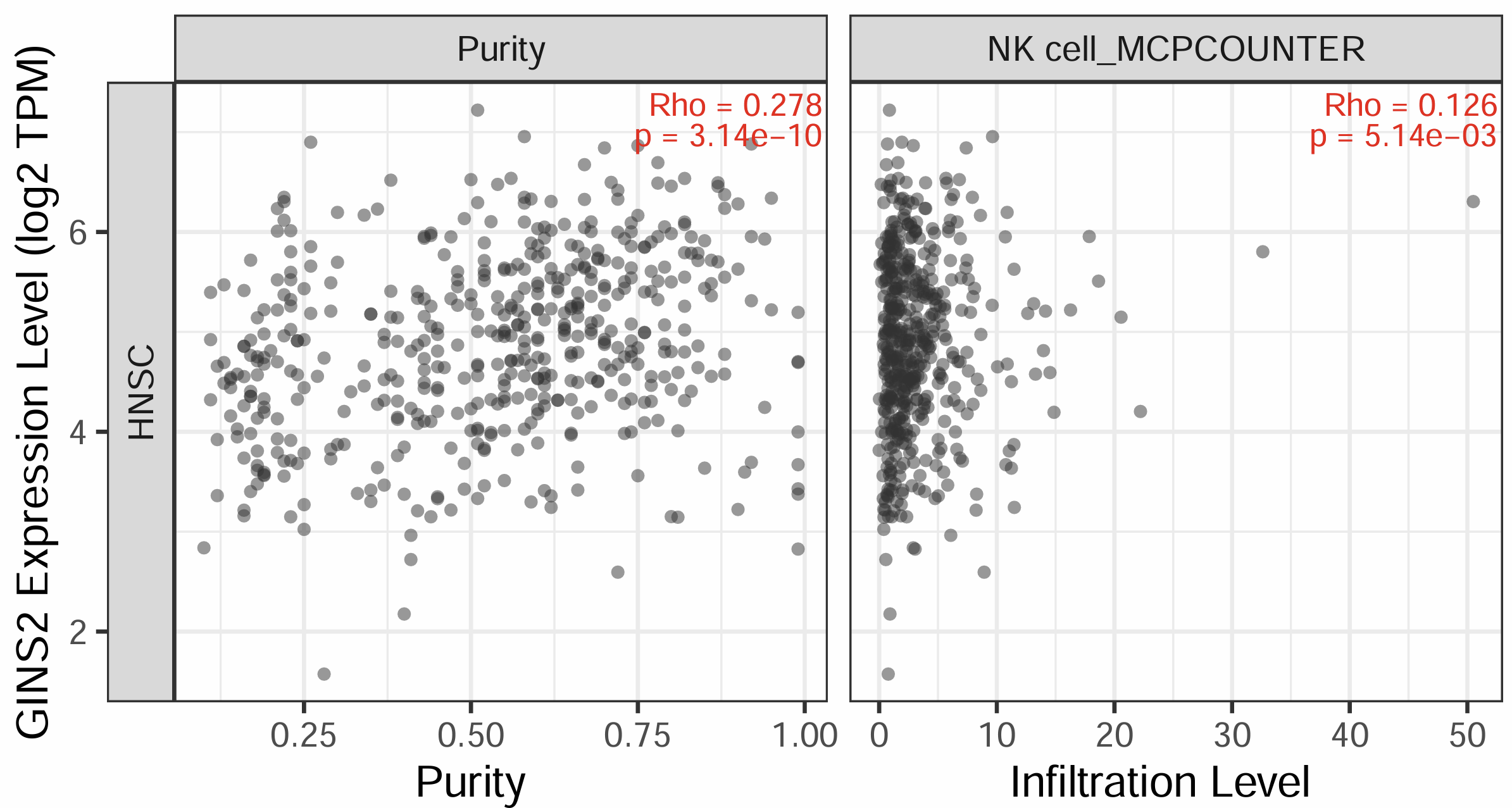
**

**Supplementary Figure S2.** **Correlation of GINS2 expression with tumor purity and NK-cell infiltration in TCGA-HNSC (TIMER2.0).** Scatterplots show GINS2 mRNA levels (log₂ TPM, y-axis) versus tumor purity (left) or NK-cell infiltration estimated by the MCP-counter algorithm (right). Each dot represents one TCGA-HNSC tumor. Spearman’s rho and two-sided P values are indicated in red. Positive rho denotes higher GINS2 expression associated with higher purity/infiltration.
